# The effect of joint contraceptive decisions on the use of Injectables, Long-Acting and Permanent Methods (ILAPMs) among married female (15–49) contraceptive users in Zambia: a cross-sectional study

**DOI:** 10.1186/1742-4755-11-51

**Published:** 2014-07-03

**Authors:** Namuunda Mutombo, Pauline Bakibinga

**Affiliations:** 1 Population Dynamics and Reproductive Health Research Program, African Population & Health Research Center, Manga Close, Off Kirawa Road, P.O. Box 10787-00100, Nairobi, Kenya; 2 Health Challenges and Systems Research Program, African Population & Health Research Center, Manga Close, Off Kirawa Road, P.O. Box 10787-00100, Nairobi, Kenya

**Keywords:** Joint contraceptive decision-making, Family planning, ILAPMs, Zambia

## Abstract

**Background:**

Zambia’s fertility rate and unmet need for family planning are still high. This is in spite of the progress reported from 1992 to 2007 of the increase in contraceptive prevalence rate from 15% to 41% and use of modern methods of family planning from 9% to 33%. However, partner disapproval of family planning has been cited by many women in many countries including Zambia. Given the effectiveness of long-acting and permanent methods of family planning (ILAPMs) in fertility regulation, this paper sought to examine the relationship between contraceptive decision-making and use of ILAPMs among married women in Zambia.

**Methods:**

This paper uses data from the 2007 Zambia Demographic and Health Survey. The analysis is based on married women (15–49) who reported using a method of family planning at the time of the survey. Out of the 7,146 women interviewed, only 1,630 women were valid for this analysis. Cross-tabulations and binary logistic regressions with Chi-square were used to analyse associations and the predictors of use of ILAPMs of contraception, respectively. A confidence interval of .95 was used in determining relationships between independent and dependent variables.

**Results:**

Two thirds of women made joint decisions regarding contraception and 29% of the women were using ILAPMs. Women who made joint contraceptive decisions are significantly more likely to use ILAPMs than women who did not involve their husband in contraceptive decisions. However, the most significant predictor is the wealth index. Women from rich households are more likely to use ILAPMs than women from medium rich and poor households. Results also show that women of North Western ethnicities and those from Region 3 had higher odds of using ILAPMs than Tonga women and women from Region 2, respectively.

**Conclusion:**

Joint contraceptive decision-making between spouses is key to use of ILAPMs in Zambia. Our findings have also shown that the wealth index is actually the strongest factor determining use of these methods. As such, family planning programmes directed at increasing use of LAPMs ought to not only encourage spousal communication but should also consider rolling out interventions that incorporate economic empowerment.

## Background

Taken as a whole, the sub-Saharan Africa region has a high (25%) unmet need for family planning [1,2]. As a result, fertility rates have not decreased significantly over the last three decades and have had negative implications on women’s sexual and reproductive health. The 1994 Cairo, International Conference on Population and Development [3] highlighted the autonomy of individuals and couples in deciding the timing, spacing and the number of children they have. Even though women’s autonomy is critical, recent evidence suggests that 10% of women with unmet need for family planning have no access to contraception owing to opposition by partner/husband [4]. This evidence suggests that there is need to include men in making contraceptive decisions. Therefore, joint contraceptive decision-making is an avenue for increasing effective contraceptive use and indeed the last decade has seen an increase in family planning literature that is rich with pronouncements about the role of spousal communication as a mechanism for increasing joint decisions about contraceptive use [5-10].

Although they are the most effective methods of modern contraception, long-acting and permanent methods (LAPMs) are the least utilised methods of contraception worldwide and especially in sub-Saharan Africa [2,11]. There are various factors responsible for this underutilisation at national, health system, provider, and couple/individual levels These factors include, inadequacy of commodities, lack of skilled providers, and the long distances that potential clients need to travel to access services, among others [2]. As such implementers of family planning programmes in the region are faced with the challenge of finding innovative ways of increasing utilization of these methods. Whereas, if consistently and effectively used, condoms (short-acting family planning method) have an added benefit of reducing the spread of HIV/AIDS, use of long acting contraceptive methods also reduces the risk of pregnancy-related death among women due to their effectiveness in reducing the incidence of unintended pregnancy; averting the likelihood of abortion and its complications. Moreover, barrier and short-term methods have high failure and discontinuation rates [12].

Contraceptive prevalence rate (CPR) for Zambia has over the years significantly increased from 15% in 1992 to 41% in 2007 and use of modern methods increased from 9% to 33% [13]. Despite this commendable progress, the level of unmet need for family planning is still high at 27% [13]. As such, Zambia still has an annual population growth rate (2.8%) that is not commensurate with the economic growth rate. According to the 2010 Zambia Census of Population and Housing, the total fertility rate is one of the highest in the Sub-Saharan Africa region, estimated at 5.9 children per woman of reproductive age. Even though Zambia has been heralded as a family planning success story, focus has been more on analysing trends on the overall CPR rather than on type of contraceptive methods. For example, the 2007 Zambia Demographic and Health Survey Report shows that the percentage of women using the intra-uterine contraceptive device (IUCD) and sterilization declined between 1992 and 2008 [14]. Factors affecting unmet need for contraception in Zambia are reported to include age, religion, region of residence, spousal communication, number of living children, and partner’s approval of family planning, among others [15].

Research on decision-making for family planning has progressively and in a timely manner moved from looking at individual roles to couples. Various studies have demonstrated that women who discuss about family planning issues with their spouses and have their partner’s approval on family planning are more likely to use a modern method of contraception [6,9,16]. A comparative analysis in 10 sub-Saharan African counties showed the positive impact of joint partner approval of family planning and discussion of family planning on a woman’s use of modern contraceptive methods [9]. Joint decisions for family planning are based on spousal communication implying that when couples communicate effectively they are more likely to jointly agree on what type of contraceptive method, how many children to have and the space between their offspring. However, in traditional Africa, decision-making regarding fertility has been dominated by male decisions [17].

The majority of the literature focussing on joint decision-making for family planning has tended to focus on contraceptive use in general [6,16,18,19]. Given that ILAPMs are the ideal choice for their cost-effectiveness and effectiveness in birth control, this report is an attempt to fill the gap in the literature by presenting the results of an investigation of the role of joint-decision making on the use of ILAPMs of contraception by married women in Zambia. The main aim of this paper is to examine the relationship between contraceptive decision-making and contraceptive method of choice among women of reproductive age in Zambia. To achieve the aim of this study we sought answers to the question, ‘Do joint contraceptive decisions lead to use of long-acting methods among married female (15–49) contraceptive users in Zambia?’.

## Data and methods

### Data source

The data for this paper were drawn from the weighted women file of the 2007 Zambia Demographic and Health Survey (ZDHS). The methodology used in this survey has been comprehensively described by the Central Statistical Office (Zambia) et al. [13]. A total of 7,146 women (15–49) and 6,500 men (15–59) were interviewed from 7,164 households using the Woman’s and Man’s Questionnaires, respectively. This paper is based on women (15–49) who reported using a method of family planning at the time of the survey. Out of the 7,146 women, only 1,069 women were valid for this analysis.

### Variables

The dependent variable used is type of contraceptive method. This variable is measured as a dichotomous variable and is coded as short-acting methods and ILAPMs. Short acting methods include all traditional and folkloric methods as well as modern methods that require some action on the part of the user just before sexual intercourse or on daily basis. The methods categorized as short acting in this paper are pill, condom (male and female), periodic abstinence, lactational amenorrhea, withdrawal and other folkloric methods. On the other hand, ILAPMs comprise of injectable substances, implants, the intra-uterine device and permanent methods. The main independent variable is the “type of contraceptive decision”. This variable is coded other if the contraceptive decision was made exclusively by respondent or partner/husband or another person, and joint if the decision was made by both respondent and partner. The study also controls for age, number of living children, ethnicity, desire for children, level of exposure to family planning messages in the media, religion, residence, level of education, wealth index, region and heard about family planning from a health worker during last 12 months.

Region was constructed by grouping the nine provinces into three groups. The grouping was based on fertility levels as reported in the 2007 ZDHS i.e. Region 1-Copperbelt and Lusaka Provinces (TFR < 5); Region 2-Central, Southern and Western Provinces (5 < TFR > 6) and Region 3-Eastern, Luapula, Northern and North Western Provinces (TFR > 6). Level of exposure to family planning messages in the media was coded none (if respondent did not hear any family planning messages in last few months); low (if respondent heard about family planning messages in last few months from only one of the three sources, namely: newspaper or radio or television), medium (if respondent heard about family planning messages in last few months from two of the three sources, namely: newspaper and radio or newspaper and television or radio and television); and high (if respondent heard about family planning messages in last few months from all the three sources, namely: newspaper, radio and television). The variable “heard about family planning from a health worker during the last 12 months” was constructed by combining information from variables V393 and V395, which show the percentage of women who were visited by a family planning health worker and the women who were told about family planning at the health facility during the last 12 months, respectively.

### Data analysis

In order to ascertain the effectiveness of joint decisions in determining the use of long-acting family planning methods among married women (15–49) in Zambia, the study used SPSS (ENTER Model) to generate frequencies, cross-tabulations and binary logistic regressions. The logit of the logistic regression model used can be expressed as follows [20,21]:

$$g\left(x\right)\;=\;\ln\left[\frac{\pi \left(x\right)}{1-\pi \left(x\right)}\right]$$

$$=\left[\pi \left(x\right)/1-\pi \left(x\right)\right]$$

$$=\;\beta_{0}+\;\beta_{1}x_{1}+\;\beta_{2}x_{2}+\;\ldots \;+\;\beta_{p}x_{p},$$

Where:

π (*x*) the odds for the dependent variable

x _1_ … *x*_p_ independent variables

β _0_ constant

β _1_ … *β*_p_ regression coefficients

The exponential β (odds ratio) was used to depict the likelihood of using ILAPMs. A ratio greater than 1 means greater likelihood of using ILAPMs than the reference category; a ratio less than 1 means lower likelihood of using ILAPMs than the reference category; and a ratio of 1 means same likelihood of using ILAPMs as the reference category. In this study, an independent variable is considered significant if its effect on the dependent variable (type of contraceptive method) is statistically significant at the .95 confidence interval (i.e. *p* ≤ 0.050). Two models are estimated: Model I fitted the main independent variable and socio-demographic variables, namely: age, number of living children and desire for children. Model II fitted all the variables fitted in Model I and socio-economic variables namely: ethnicity, level of exposure to family planning messages in the media, religion, residence, level of education, wealth index, region and heard about family planning from a health worker during last 12 months.

## Results

### Characteristics of the study population

Table 1 shows the characteristics of the study respondents. Most (82%) women in this sample were non-Catholic while approximately two thirds of women said that they made joint decisions regarding contraception. Almost half (48%) were of the middle reproductive ages (25–34). According to number of children, a large proportion (37%) of women had three or four children while slightly more than half (58%) of women said they wanted to have another child in future but no future period was mentioned. Bemba-speaking women constituted the largest (36%) single ethnic grouping and majority (40%) of women were from Region 3, which includes the predominantly Bemba-speaking provinces (Luapula and Northern).

**Table 1:** Percentage distribution of women (15–49) included in the analysis by selected characteristics in Zambia

**Characteristics**	**%**	**n**
*Type of contraceptive decision*		
Other	34.2	366
Joint	65.8	703
*Age*		
15-24	24.9	266
25-34	48.3	516
35-49	26.8	287
*Number of living children*		
≤2	33.0	353
3-4	37.0	396
5+	30.0	320
*Ethnicity*		
Bemba	35.7	382
Lozi	10.3	110
North Westerner (Kaonde, Lunda & Luvale)	10.7	114
Nyanja-speaking	29.0	310
Tonga	14.3	153
*Desire for children*		
Wants another	57.7	617
Undecided	5.5	59
Wants no more	36.8	393
*Level of exposure to family planning messages in the media*		
None	47.7	510
Low	28.1	300
Medium	13.8	147
High	10.4	112
*Heard about FP from a health worker during last 12 months*		
No	34.6	370
Yes	65.4	699
*Religion*		
Catholic	17.6	188
Non-Catholic	82.4	881
*Residence*		
Urban	46.0	492
Rural	54.0	577
*Level of education*		
None	9.4	101
Primary	54.7	585
Secondary+	35.9	383
*Wealth index*		
Poor	32.7	350
Medium	17.1	183
Rich	50.2	536
*Region*		
Region 1 (Copperbelt & Lusaka)	31.5	337
Region 2 (Central, Southern & Western)	28.9	309
Region 3 (Eastern, Luapula, Northern & North Western	39.6	423
*Type of contraceptive method*		
Short-acting	70.5	754
Long-acting	29.5	315
**Total**	**100.0**	**1,069**

By residence, majority (54%) of the women were from some rural dwelling while women from rich households constituted half of the sample. Education levels are generally low as slightly more than a third of the women had attained education higher than primary level. However, more than half of all the women had heard a family planning message in the media in recent months. The data also reveal some moderate level of interaction with health workers as sources of family planning information in Zambia. In this study, 65% of the women reported having heard about family planning from a health worker within 12 months prior to their interview date. However, less than one third of these women were using ILAPMs.

### Prevalence of ILAPMs use

Approximately 30% of the 1,069 women reported using LAPMs. The results in Table 2 show some significant associations between type of contraceptive method and some characteristics and these characteristics are discussed below.

**Table 2:** Prevalence of ILAPMs by women’s (15–49) selected characteristics in Zambia

**Background characteristics**	**Type of contraceptive method**		**Total**
	**Short-acting**	**ILAPMs**	
Type of contraceptive decision**			
Other	76.0	24.0	100.0
Joint	69.1	30.9	100.0
Age**			
15-24	74.7	25.3	100.0
25-34	73.5	26.5	100.0
35-49	66.1	33.9	100.0
Number of living children**			
≤2	74.7	25.3	100.0
3-4	73.8	26.2	100.0
5+	66.1	33.9	100.0
Ethnicity***			
Bemba	72.1	27.9	100.0
Lozi	78.0	22.0	100.0
North Westerner (Kaonde, Lunda & Luvale)	57.8	42.2	100.0
Nyanja	73.6	26.4	100.0
Tonga	72.4	27.6	100.0
Desire for children***			
Wants another	75.2	24.8	100.0
Undecided	76.2	23.8	100.0
Wants no more	66.0	34.0	100.0
Level of exposure to FP messages in the media**			
None	74.3	25.7	100.0
Low	72.4	27.6	100.0
Medium	67.3	32.7	100.0
High^†^	63.1	36.9	100.0
*Heard about FP from a health worker during last 12 months*			
No	70.8	29.2	100.0
Yes	70.8	29.2	100.0
Religion*			
Catholic	77.0	23.0	100.0
Non-Catholic	70.5	29.5	100.0
Residence**			
Urban	67.5	32.5	100.0
Rural	75.0	25.0	100.0
Level of education*			
None	77.4	22.6	100.0
Primary	72.5	27.5	100.0
Secondary or higher	68.5	31.5	100.0
Wealth index***			
Poor	78.6	21.4	100.0
Medium	74.2	25.8	100.0
Rich	66.1	33.9	100.0
*Region*			
Region 1 (Copperbelt & Lusaka)	71.6	28.4	100.0
Region 2 (Central, Southern & Western)	72.7	27.3	100.0
Region 3 (Eastern, Luapula, Northern & North Western	71.1	28.9	100.0

*p < 0.050; **p < 0.010; ***p < 0.001.

Though not the strongest association, women who made joint contraceptive decisions had a higher (31%) prevalence of LAPMs use than women whose decisions were not made jointly with husband (24%). The most significant associations are observed by desire for children, ethnicity and the wealth index. Women of North Western (Kaonde, Lunda & Luvale) ethnicities have the highest percentage (42%) of use of ILAPMs than women from other ethnic groups. As expected, women who did not want to have any more children reported the highest (34%) use of ILAPMs than the women who wanted another child (25%) or were undecided about having another child (24%). The data also exhibit some glaring differences by the household wealth index of a woman. Women from rich households had the highest (34%) use of ILAPMs of contraception, followed by women from the medium wealth household category (26%). Women from poor women had the lowest (22%) use of ILAPMs.

Other variables with significant associations, though not as strong as wealth index, ethnicity and desire for children, are age, number of children, level of exposure to family planning messages in the media, religion, residence and level of education. Women in their late reproductive ages (35+) have the highest use of ILAPMs (34%) when compare with middle aged and younger women. Non-Catholics have higher (30%) use of ILAPMs of contraception than Catholic women (24%) while urban women have higher (33%) prevalence of ILAPMs than their rural counterparts (25%). Table 2 also shows that women with five or more children had the highest use of ILAPMs (34%) compare with women with fewer than five children. Access to family planning messages in the media also shows significant associations with use of ILAPMs as the proportion of ILAPMs users increases as level of exposure to family planning in the media increases. A similar observation is also made for education.

### Predictors of ILAPMs use

Two models are used to present the results of the determinants of use of ILAPMs (Table 3). The odds ratio is used to depict the likelihood of using ILAPMs and the interpretation of the odds ratio has already been provided in the methods section of this. Results of Model I show that type of contraceptive decision was statistically significant on the choice of contraceptive method. Women who made contraceptive decisions without involving their spouses were 30% less likely to be using ILAPMs than women who made joint decisions. Model I also shows that use of ILAPMs varies significantly by a woman’s desire for children. Women who said they wanted to have another child were significantly less likely to use ILAPMs than those who said they did not want to have any more children. However, there was no significant difference between women who were undecided about having additional children and women who did not want to have any more children.

**Table 3:** Odds ratios of using ILAPMs among current contraceptive users in Zambia by selected characteristics

**Background characteristics**	**Model**	
	**I**	**II**
*Type of contraceptive decision*		
Other	0.709**	0.724*
Joint^†^	-	-
*Age*		
15-24	0.924	1.353
25-34	0.907	1.065
35-49^†^	-	-
*Number of living children*		
≤2	0.846	0.695
3-4	0.816	0.745
5 +^†^	-	-
*Desire for children*		
Wants another	0.707*	0.829
Undecided	0.668	0.938
Wants no more^†^	-	-
*Ethnicity*		
Bemba	-	1.259
Lozi	-	1.074
North Westerner (Kaonde, Lunda & Luvale)	-	2.183*
Nyanja	-	1.350
Tonga^†^	-	-
*Level of exposure to FP messages in the media*		
None	-	0.694
Low	-	0.711
Medium	-	0.620
High^†^	-	-
*Heard about FP from a health worker during last 12 months*		
No	-	0.930
Yes^†^	-	-
*Religion*		
Catholic	-	0.823
Non-Catholic^†^	-	-
*Residence*		
Urban	-	0.921
Rural^†^	-	-
*Level of education*		
None	-	0.817
Primary	-	1.137
Secondary or higher^†^	-	-
*Wealth index*		
Poor	-	0.443**
Medium	-	0.598*
Rich^†^	-	-
*Region*		
Region 1 (Copperbelt & Lusaka)	-	0.741
Region 2 (Central, Southern & Western)	-	0.640*
Region 3^†^ (Eastern, Luapula, Northern & North Western	-	-

^†^Reference category; *p < 0.050; **p < 0.010.

In Model II, we added the socio-economic variables and some significant changes to the multivariate results in Model I are noteworthy. Though women who made joint decisions on family planning had significantly higher odds of using ILAPMs than women whose decision was not made with husband, the strength of this relationship is weaker in Model II than in Model I and the desire for children is insignificant in Model II. Some significant differences are also observed by ethnicity, particularly between the Tonga women (reference group) and women of North Western ethnic groups (Kaonde, Lunda and Luvale). However, there are no significant differences in use of ILAPMs between women of other ethnicities (Bemba-speaking, Lozi and Nyanja-speaking) and Tonga women.

Another significant predictor of long-acting contraception is region. Women in Regions 2 have significantly lower odds of using ILAPMS than women in Region 3 while the likelihood of using ILAPMs among women in Region 1 is also higher than among women in Region 3 but is statistically not significant. However, the strongest predictor of use of long-acting methods among Zambian women is the wealth index. Women from poor households are approximately 56% less likely to use ILAPMs than women from rich households while women from medium wealthy households category are 36% less likely to use ILAPMs than women from rich households.

## Discussion

This paper sought to examine the effect of joint contraceptive decisions on use of ILAPMs. A number of control variables were also included in order to assess the net effect of joint contraceptive decisions. Results indicate that majority of the married women in Zambia make joint contraceptive decisions (i.e. with partner/husband). This finding is consistent with the available literature stressing thatI LAPMs are the least utilised methods of contraception [2,11,22]. Results from our analyses also underscore the important finding that women who involve their partners in making decisions regarding family planning are more likely to use ILAPMs than women who do not make contraceptive decisions with their partners. This observation is key to unlocking women’s access to contraceptive technologies as many studies show that partner approval is quite often a precondition for women’s use of contraceptives [6,16]. For example, a recent review of data from South East Asia, South Central Asia and Sub-Saharan Africa among women with unmet need for modern contraceptives showed that 10% of them reported opposition from partner [4].

However, joint decision- making is not the strongest predictor for the use of ILAPMs of contraception among married women in Zambia. The strongest predictor is the wealth index. Women from wealthy households have higher odds of using ILAPMs than women from poor and moderately wealthy households. This observation is in a way in tandem with the wealth flow theory of fertility. According to Kaplan [23], people will desire more children when net wealth transfer from children to parents is positive and people will desire fewer children when there is negative wealth flow from children to parents. This theory is to a large extent applicable to Zambia when it comes to fertility regulation as children are also a source of labour in a largely agrarian society but with a poorly mechanized agricultural sector. Based on these results, we speculate that economic empowerment of households in Zambia will reduce the economic expectations of parents from children. Wealth has previously been reported as a contributor to contraceptive use among women in developing countries, with wealthier women having more access to and utilisation of birth control measures [24,25]. Crissman [24] found that increasing wealth was one of the main factors associated with contraceptive use among non-pregnant married and partnered women who did not want to get pregnant in the next three months in Ghana. These past findings and our results could also be attributed to challenges of access due to financial constraints by poor women [25].

Our findings also show that the odds of using ILAPMs are also high among women from Region 3 (Eastern, Luapula, Northern and North Western Provinces) and women of North Western ethnicities. It is, however, not very clear as to what factors are responsible for this observation but the United Nations Population Fund (UNFPA)’s regional program approach appears to be influential on use of long acting methods for both Region 3 and women of North Western ethnic groups. UNFPA has been implementing a reproductive health program in the North Western Province since 2002 and extended this program to Luapula Province in 2007. One of the key interventions in this program has been the provision of ILAPMs.

Furthermore, and as expected, women who desire another child are less likely to use of LAPMs than women who are undecided or do not want to have another child. Relatedly, women with fewer than five children are also less likely to use ILAPMs than those with five or more children. This finding is in tandem with the observation by Bessinger et al. [26] that Zambian women prefer an average of five children. Since long-acting methods are very effective and coupled with myths about some side effects, women who have not attained this family size are generally less likely to use ILAPMs. Age of respondent and level of education, religion and urban/rural residence have also been identified as important contributors to unmet need for contraception and contraceptive use [15,27]. However, our analysis showed that age and education were not significant. Similarly, that although Catholic and urban women showed lower odds of using ILAPMs, the results were not significant.

Finally, our analysis further revealed that level of exposure to family planning messages in the media (newspaper, radio and television) in recent months appeared to be inconsequential on the odds of using ILAPMs of family planning just like hearing about family planning from a health worker within 12 months prior to the survey has no significance on use of long-acting contraceptives. This is contrary to some of the family planning literature on patterns of contraceptive use that has shown that a visit by a family planning worker and/or exposure to mass media messages was a significant contributor to a woman’s use of long-term methods [28].

### Limitations

Within the context of these findings, caution is needed in the interpretation of the results of this study as it uses cross-sectional survey data. As such causal relationships between variables and outcomes cannot be accounted for, only associations. Lack of qualitative data to complement these results accentuates this limitation. There are several factors influencing access to and use of family planning products such as availability of contraceptives that are not included in DHS data, about which this paper can only speculate.

## Conclusion

This paper has demonstrated that joint contraceptive decisions between spouses is important to use of ILAPMs, which are the most effective methods of fertility regulation. Increasing access to ILAPMs will also help reduce maternal deaths as well as control population growth. Our findings have also shown that even though joint-decision making is a key predictor of use of ILAPMs of contraception among married women in Zambia, the wealth index is actually the strongest factor determining use of these methods. As such, family planning programmes directed at increasing use of ILAPMs ought not only encourage spousal communication, hence joint-decision making, but also to consider and roll- out interventions that incorporate economic empowerment for households. This will serve to increase uptake and utilisation of ILAPMs of contraception among economically disadvantaged groups.

## Competing interests

The authors declare that there are no competing interests.

## Authors’ contributions

This work was a collaborative effort between both authors. NM conceptualised the study, ran the statistical analysis and wrote part of the first draft of the manuscript. PB took part in the interpretation of the data, managed the literature searches and wrote part of the first draft of the manuscript. Both authors read and approved the final manuscript.

## Authors’ information

NM is a demographer, PhD, formerly with Ministry of Finance, Zambia and the University of Zambia; currently a researcher with the African Population and Health Research Center, Nairobi, Kenya.

PB is a medical doctor, PhD, formerly worked as a clinician and researcher in Uganda; currently a researcher with the African Population and Health Research Center, Nairobi, Kenya.
